# Emergence and Characterization of Unusual DS-1-Like G1P[8] Rotavirus Strains in Children with Diarrhea in Thailand

**DOI:** 10.1371/journal.pone.0141739

**Published:** 2015-11-05

**Authors:** Satoshi Komoto, Ratana Tacharoenmuang, Ratigorn Guntapong, Tomihiko Ide, Kei Haga, Kazuhiko Katayama, Takema Kato, Yuya Ouchi, Hiroki Kurahashi, Takao Tsuji, Somchai Sangkitporn, Koki Taniguchi

**Affiliations:** 1 Department of Virology and Parasitology, Fujita Health University School of Medicine, Toyoake, Aichi, Japan; 2 Department of Medical Sciences, National Institute of Health, Nonthaburi, Thailand; 3 Department of Virology II, National Institute of Infectious Diseases, Musashi-Murayama, Tokyo, Japan; 4 Division of Molecular Genetics, Institute for Comprehensive Medical Science, Fujita Health University, Toyoake, Aichi, Japan; 5 Genome and Transcriptome Analysis Center, Fujita Health University, Toyoake, Aichi, Japan; 6 Department of Microbiology, Fujita Health University School of Medicine, Toyoake, Aichi, Japan; University of Hong Kong, HONG KONG

## Abstract

The emergence and rapid spread of unusual DS-1-like G1P[8] rotaviruses in Japan have been recently reported. During rotavirus surveillance in Thailand, three DS-1-like G1P[8] strains (RVA/Human-wt/THA/PCB-180/2013/G1P[8], RVA/Human-wt/THA/SKT-109/2013/G1P[8], and RVA/Human-wt/THA/SSKT-41/2013/G1P[8]) were identified in stool specimens from hospitalized children with severe diarrhea. In this study, we sequenced and characterized the complete genomes of strains PCB-180, SKT-109, and SSKT-41. On whole genomic analysis, all three strains exhibited a unique genotype constellation including both genogroup 1 and 2 genes: G1-P[8]-I2-R2-C2-M2-A2-N2-T2-E2-H2. This novel genotype constellation is shared with Japanese DS-1-like G1P[8] strains. Phylogenetic analysis revealed that the G/P genes of strains PCB-180, SKT-109, and SSKT-41 appeared to have originated from human Wa-like G1P[8] strains. On the other hand, the non-G/P genes of the three strains were assumed to have originated from human DS-1-like strains. Thus, strains PCB-180, SKT-109, and SSKT-41 appeared to be derived through reassortment event(s) between Wa-like G1P[8] and DS-1-like human rotaviruses. Furthermore, strains PCB-180, SKT-109, and SSKT-41 were found to have the 11-segment genome almost indistinguishable from one another in their nucleotide sequences and phylogenetic lineages, indicating the derivation of the three strains from a common origin. Moreover, all the 11 genes of the three strains were closely related to those of Japanese DS-1-like G1P[8] strains. Therefore, DS-1-like G1P[8] strains that have emerged in Thailand and Japan were assumed to have originated from a recent common ancestor. To our knowledge, this is the first report on whole genome-based characterization of DS-1-like G1P[8] strains that have emerged in an area other than Japan. Our observations will provide important insights into the evolutionary dynamics of emerging DS-1-like G1P[8] rotaviruses.

## Introduction

Group A rotavirus (RVA), a member of the *Reoviridae* family, is the most important etiological agent of severe gastroenteritis in infants and young children. RVA infections are associated with high morbidity and mortality, being responsible for an estimated 453,000 deaths each year in children <5 years of age [1]. The RVA virion is a non-enveloped, triple-layered icosahedron that encapsidates an 11-segment genome of double-stranded (ds)RNA [2]. Due to the segmented nature of the genome, reassortment between/among RVAs is one of the major processes of genetic evolution of this medically important virus.

RVA has two outer capsid proteins, VP7 and VP4, which are implicated independently in neutralization, and define the G and P genotypes, respectively. To date, RVAs have been classified into at least 27 G and 37 P genotypes [3, 4]. Among them, 5 G (G1-4, G9, and G12) and 3 P (P[4], P[6], and P[8]) genotypes are commonly associated with RVA disease in humans [5, 6].

A whole genome-based genotyping system was recently proposed for RVAs based on the assignment to all the 11 gene segments (i.e., G/P and non-G/P genes) [7]. In the new genotyping system, the acronym Gx-P[x]-Ix-Rx-Cx-Mx-Ax-Nx-Tx-Ex-Hx, where x is an integer, defines the genotype of the VP7-VP4-VP6-VP1-VP2-VP3-NSP1-NSP2-NSP3-NSP4-NSP5 genes of a given RVA strain. Most human RVA strains have genes similar in sequence to those of prototype human strains Wa (genogroup 1 genes) or DS-1 (genogroup 2 genes) [8]. The Wa-like strains are characterized by non-G/P genotypes (I1-R1-C1-M1-A1-N1-T1-E1-H1), and tend to have G/P genotypes G1P[8], G3P[8], G4P[8], G9P[8], G12P[6], and G12P[8] [9]. In contrast, the DS-1-like strains are characterized by non-G/P genotypes (I2-R2-C2-M2-A2-N2-T2-E2-H2), and tend to have G/P genotypes G2P[4]. There have been occasional reports on the identification of intergenogroup reassortants between the genogroup 1 and 2 genes [10]. Although intergenogroup reassortant can exist, it is believed that such RVA strains possessing both genogroup 1 and 2 genes have a decreased evolutionary fitness compared to the parental strains, and would be selected against in the context of infection in the human population [8, 11]. However, the emergence and rapid spread of novel human intergenogroup reassortant strains possessing DS-1-like non-G/P genotypes (I2-R2-C2-M2-A2-N2-T2-E2-H2) with G/P genotypes G1P[8]: G1-P[8]- I2-R2-C2-M2-A2-N2-T2-E2-H2, which had never been previously described, have been recently reported in several districts in Japan (DS-1-like G1P[8] strains) [10, 12, 13].

The first DS-1-like G1P[8] strains, including strain OH3506, were detected in children with acute diarrhea in Okayama Prefecture, Japan in 2012 [12], and subsequently such intergenogroup reassortant strains, including strains HC12016 and NT004, were identified in Aichi, Akita, Kyoto, and Osaka Prefectures, Japan in 2012–2013 [10, 13]. Notably, DS-1-like G1P[8] strains became wide spread in the human population and were predominant in several separate locations in Japan. In 2013, we detected three DS-1-like G1P[8] strains, PCB-180, SKT-109, and SSKT-41, in diarrheic children in Phechaboon and Sukhothai Provinces, Thailand, a total of 687 RVA-positive stool specimens being examined by PCR-based G and P genotyping, and polyacrylamide gel electrophoresis (PAGE) analysis (Tacharoenmuang et al., in preparation). To our knowledge, strains PCB-180, SKT-109, and SSKT-41 are the first DS-1-like G1P[8] strains that have emerged in an area other than Japan.

Whole genome-based analysis is a reliable method for obtaining conclusive data on the origin of an RVA strain, and for tracing its evolutionary pattern [7, 14]. To date, the whole genome sequences of several Japanese DS-1-like G1P[8] strains have been fully sequenced and characterized, which indicated the occurrence of reassortment event(s) between Wa-like G1P[8] and DS-1-like human rotaviruses [10, 13]. Furthermore, Japanese DS-1-like G1P[8] strains were very closely related to one another in all the 11 genes, indicating that these Japanese strains are epidemiologically linked to one another [10]. However, as the overall genomic constellation and the genomic diversity of DS-1-like G1P[8] strains remain to be elucidated, whole genomic analysis of the Thai DS-1-like G1P[8] strains might be useful for obtaining a more precise understanding of the evolutionary pattern of DS-1-like G1P[8] strains. In this study, deep sequencing with the next generation sequencing (NGS) Illumina MiSeq platform was performed to obtain the complete nucleotide sequences of the whole genomes of these three Thai DS-1-like G1P[8] strains.

## Materials and Methods

### Ethics statement

The study was approved by the Ethical Review Committee for Research in Human Subjects of the Ministry of Public Health, Thailand (Ref. no. 10/2555). In this study, written informed consent for the testing of stool samples for RVAs and characterization of identified RVA strains was obtained from the children’s parents/guardians.

### Virus strains

The full-genomic sequences were determined for strains PCB-180, SKT-109, and SSKT-41, which were identified in three fecal specimens from hospitalized children aged 16–49 months with severe diarrhea in Phechaboon and Sukhothai Provinces during the RVA surveillance program in Phechaboon and Sukhothai Provinces, Thailand in 2012–2014, which involved a total of 3002 stool samples (Tacharoenmuang et al., in preparation). Of the 3002 stool specimens, RVA infection was detected in 687 (22.9%). Stool samples were collected during hospital-based surveillance activities under the RVA vaccine effectiveness evaluation study in Thailand. Stool samples containing strains PCB-180, SKT-109, and SSKT-41 were kept at −30°C until use.

### Viral dsRNA extraction

The viral dsRNAs were extracted from fecal suspensions using a QIAamp Viral RNA Mini Kit (Qiagen). The extracted dsRNAs were used for (i) polyacrylamide gel electrophoresis (PAGE) analysis, and (ii) whole genomic analysis. For PAGE analysis, the dsRNAs were electrophoresed in a 10% polyacrylamide gel for 16 h at 20 mA at room temperature, followed by silver staining [15] to determine the genomic dsRNA profile. For whole genomic analysis, viral dsRNAs were subjected to Illumina MiSeq sequencing as described below.

### cDNA library building and Illumina MiSeq sequencing

Preparation of a cDNA library and Illumina MiSeq sequencing were performed as described previously [9, 16–18]. Briefly, a 200 bp fragment library ligated with bar-coded adapters was constructed for strains PCB-180, SKT-109, and SSKT-41 using NEBNext Ultra RNA Library Prep Kit for Illumina v1.2 (New England Biolabs) and NEBNext Multiplex Oligos for Illumina (Index Primers Set 1) (New England Biolabs) according to the manufacturer’s instructions. Library purification was carried out using Agencourt AMPure XP magnetic beads (Beckman Coulter). The quality of the purified cDNA library was assessed on an Agilent 2100 Bioanalyzer (Agilent Technologies). Nucleotide sequencing was performed on an Illumina MiSeq sequencer (Illumina) using a MiSeq Reagent Kit v2 (Illumina) to generate 151 paired-end reads. Data analysis was performed using CLC Genomics Workbench v8.0.1 (CLC Bio). Contigs were assembled from the obtained sequence reads by *de novo* assembly. Using the assembled contigs as query sequences, the Basic Local Alignment Search Tool (BLAST) non-redundant nucleotide database was searched to obtain the full-length nucleotide sequence of each gene segment of strains PCB-180, SKT-109, and SSKT-41. The nucleotide sequences were translated into amino acid sequences using GENETYX v11 (GENETYX).

### Determination of RVA genotypes

The genotype of each of the 11 gene segments of strains PCB-180, SKT-109, and SSKT-41 was determined using the RotaC v2.0 automated genotyping tool (http://rotac.regatools.be/) [19] according to the guidelines proposed by the Rotavirus Classification Working Group (RCWG).

### Phylogenetic analyses

Sequence comparisons were carried out as described previously [17, 20]. Briefly, multiple alignment of each viral gene was performed using CLUSTAL W [21]. Phylogenetic trees were constructed using the maximum likelihood method and the Tamura-Nei substitution model using MEGA6.06 [22]. The reliability of the branching order was estimated from 1000 bootstrap replicates [23].

### Nucleotide sequence accession numbers

The nucleotide sequence data presented in this manuscript have been deposited in the DDBJ and EMBL/GenBank data libraries. The accession numbers for the nucleotide sequences of the VP1-4, VP6-7, and NSP1-5 genes of strains PCB-180, SKT-109, and SSKT-41 are LC066639-LC066649, LC066650-LC066660, and LC066661-LC066671, respectively.

## Results and Discussion

### Profiles of genomic dsRNAs of strains PCB-180, SKT-109, and SSKT-41 on PAGE

**Fig 1:**
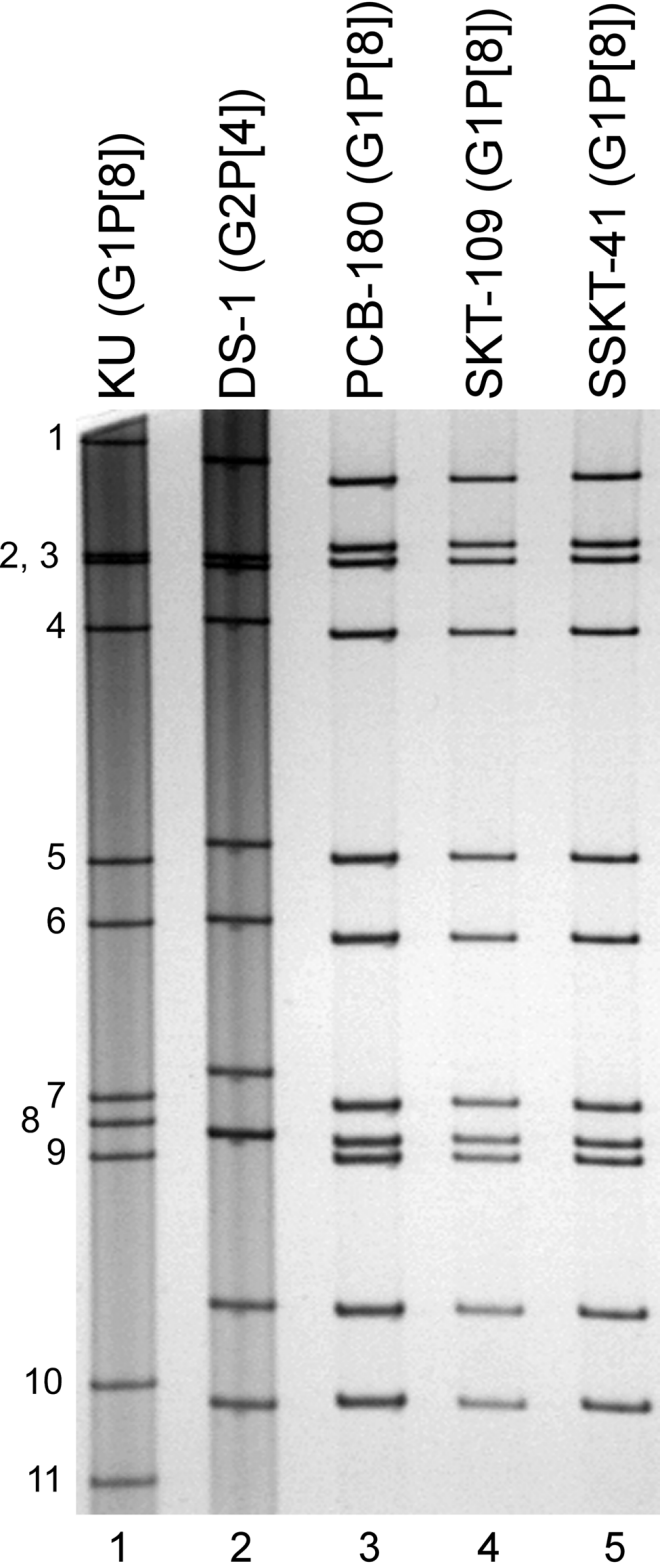
Genomic dsRNA profiles of strains PCB-180, SKT-109, and SSKT-41. Lanes 1–2, dsRNAs of strains KU (G1P[8]) (lane 1) and DS-1 (G2P[4]) (lane 2) extracted from the cell cultures; and lanes 3–5, dsRNAs of strains PCB-180 (lane 3), SKT-109 (lane 4), and SSKT-41 (lane 5) extracted from stool samples. The numbers on the left indicate the order of the genomic dsRNA segments of strain KU.

The virion dsRNAs of strains PCB-180, SKT-109, and SSKT-41 were extracted from stool samples and then analyzed by PAGE. Fig 1 shows the profiles of viral dsRNAs from strains PCB-180 (lane 3), SKT-109 (lane 4), and SSKT-41 (lane 5) from fecal samples. Instead of the long electropherotype found in most Wa-like strains, strains PCB-180, SKT-109, and SSKT-41 had the short electropherotype found in most DS-1-like strains. Of note was that strains PCB-180, SKT-109, and SSKT-41 had an almost identical electropherotype, suggesting a close genetic relatedness among the three strains.

### Nucleotide sequencing and whole-genome-based genotyping of strains PCB-180, SKT-109, and SSKT-41

In order to gain an insight into the genetic variability among strains PCB-180, SKT-109, and SSKT-41, and the genetic relatedness with other RVA strains worldwide, the full-genome sequences of all the 11 segments of these three strains were determined using the NGS Illumina MiSeq platform. The whole genomes of the three strains were amplified using a sequence-independent primer set and then sequenced successfully. Illumina MiSeq sequencing yielded 14 x 10^5^ reads (~121 bp average length), 8.5 x 10^5^ reads (~136 bp average length), and 11 x 10^5^ reads (~131 bp average length) for strains PCB-180, SKT-109, and SSKT-41, respectively. Complete or nearly complete nucleotide sequences of all the 11 segments of the three strains could be obtained. The lengths of nucleotide and deduced amino acids sequences of the 11 gene segments of strains PCB-180, SKT-109, and SSKT-41, with related sequence read data is summarized in S1 Table.

**Fig 2:**
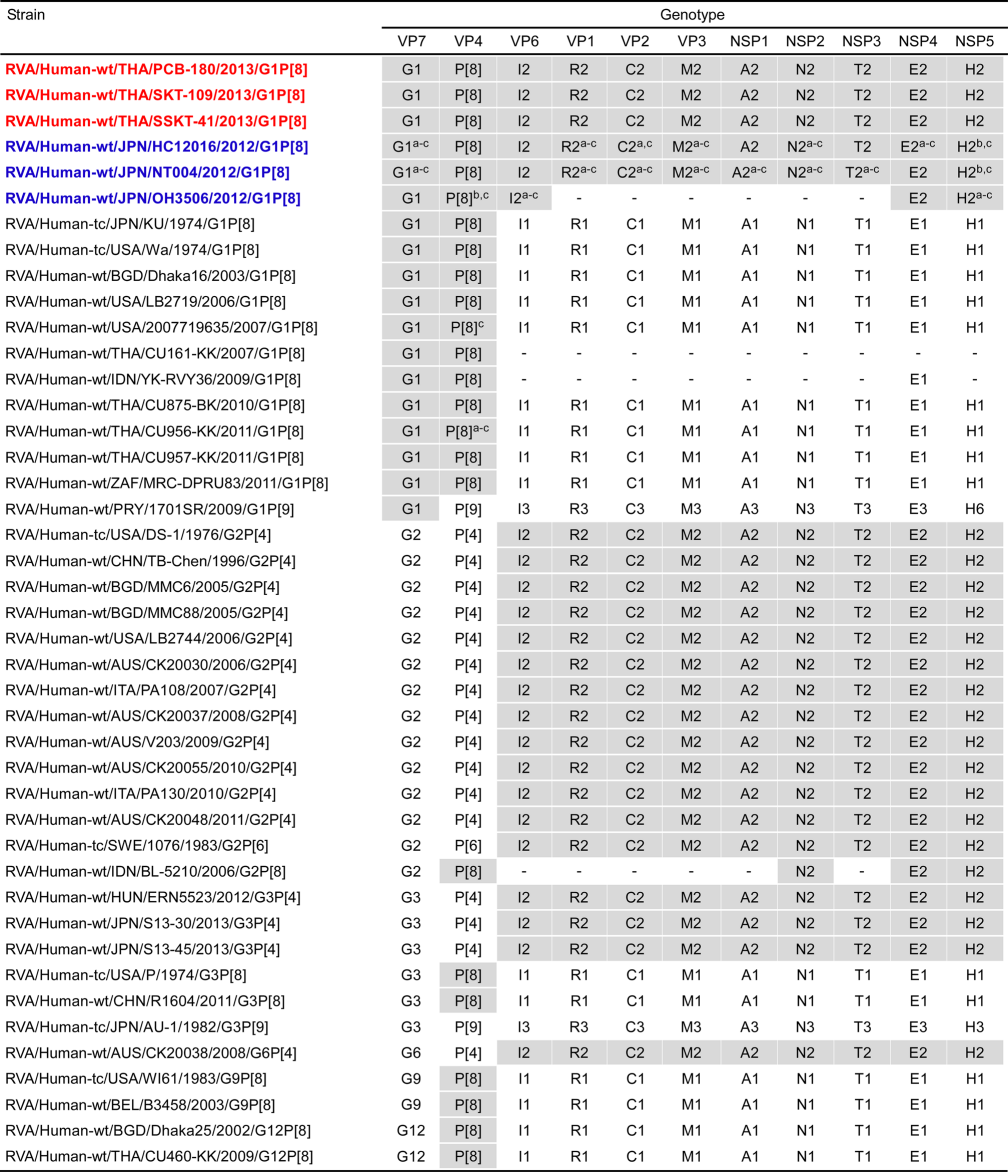
Genotype natures of the 11 gene segments of three Thai DS-1-like G1P[8] strains, PCB-180, SKT-109, and SSKT-41, compared with those of selected human strains. Strains PCB-180, SKT-109, and SSKT-41 are shown in red, while other DS-1-like G1P[8] strains are shown in blue. Gray shading indicates the gene segments with genotypes identical to those of strains PCB-180, SKT-109, and SSKT-41. “−” indicates that no sequence data were available in the DDBJ and EMBL/GenBank data libraries. ^a^The gene segments that are most similar to those of strain PCB-180. ^b^The gene segments that are most similar to those of strain SKT-109. ^c^The gene segments that are most similar to those of strain SSKT-41.

The 11 genes of strains PCB-180, SKT-109, and SSKT-41 were all assigned as G1-P[8]-I2-R2-C2-M2-A2-N2-T2-E2-H2 (Fig 2). Strains PCB-180, SKT-109, and SSKT-41 were confirmed to have G1P[8] genotypes and a DS-1-like genetic backbone, as indicated on PCR-based G and P genotyping, and electropherotyping, respectively (Tacharoenmuang et al., in preparation). Thus, strains PCB-180, SKT-109, and SSKT-41 were named RVA/Human-wt/THA/PCB-180/2013/G1P[8], RVA/Human-wt/THA/SKT-109/2013/G1P[8], and RVA/Human-wt/THA/SSKT-41/2013/G1P[8], respectively, according to the guidelines for the uniformity of RVAs proposed by the RCWG. Comparison of the complete genotype constellations of strains PCB-180, SKT-109, and SSKT-41 with those of other G1P[8] and non-G1P[8] strains is shown in Fig 2. Strains PCB-180, SKT-109, and SSKT-41 had a DS-1-like non-G/P genotype constellation (I2-R2-C2-M2-A2-N2-T2-E2-H2), which is commonly found in Japanese DS-1-like G1P[8] strains [11, 13]. Thus, the complete genotype constellation of strains PCB-180, SKT-109, and SSKT-41 (G1-P[8]-I2-R2-C2-M2-A2-N2-T2-E2-H2) is identical to that of Japanese DS-1-like G1P[8] strains. Furthermore, as suggested by the genomic dsRNA profiles observed on PAGE analysis (Fig 1), strains PCB-180, SKT-109, and SSKT-41 exhibited extremely high nucleotide sequence identities (99.7–100%) to one another for all the 11 gene segments (data not shown).

### Phylogenetic analyses

We next constructed phylogenetic trees using the full-genome sequence for each of the 11 gene segments because phylogenetic analysis of RVA nucleotide sequences provides direct evidence of their relatedness to those of other strains, even within the same genotype [7]. In this study, we employed strains HC12016 [13], NT004 [10], and OH3506 [12] as representative Japanese DS-1-like G1P[8] strains, which were analyzed in three independent studies in 2014.

**Fig 3:**
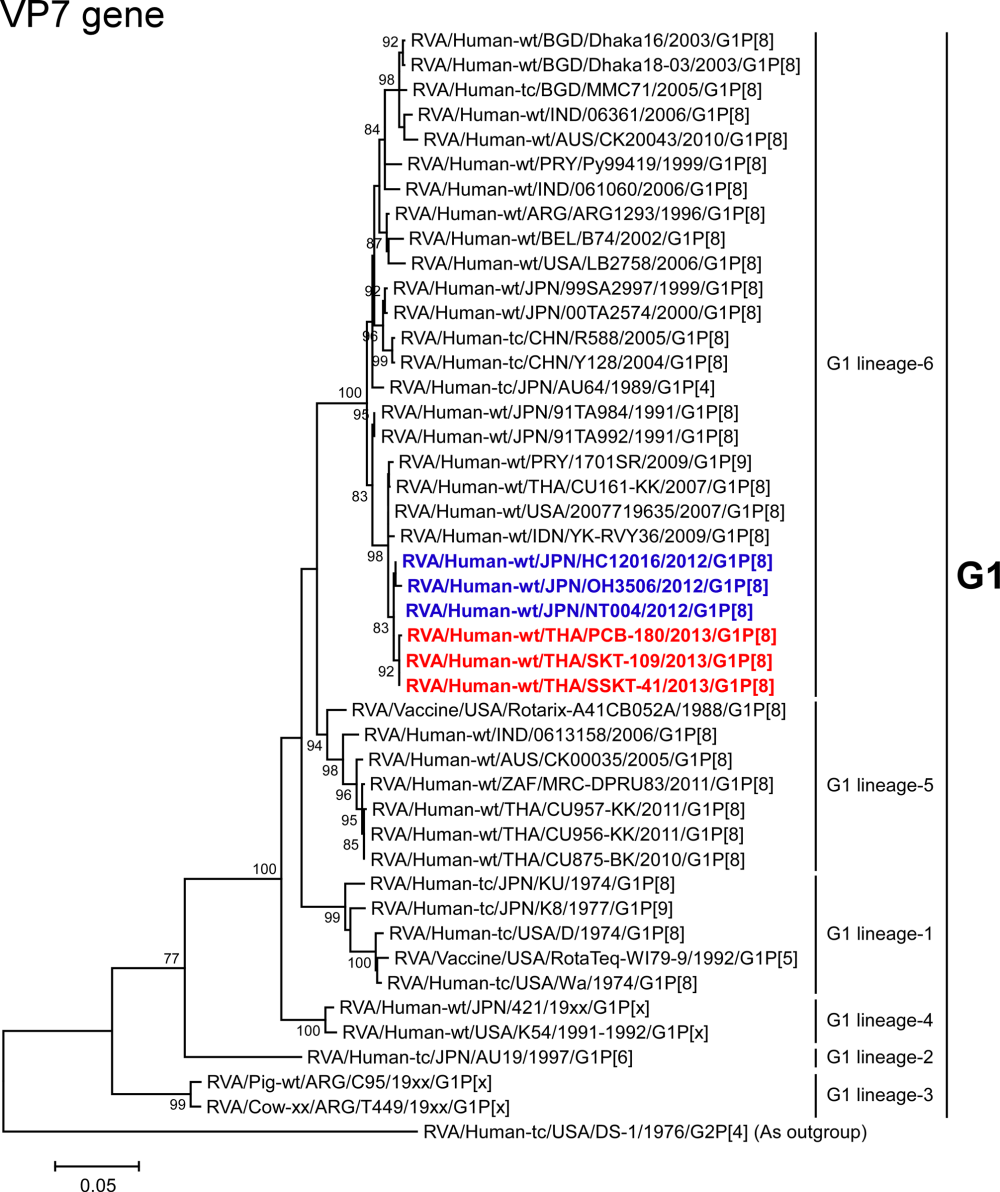
Phylogenetic tree constructed from the nucleotide sequences of the VP7 genes of strains PCB-180, SKT-109, and SSKT-41, and representative RVA strains. In the tree, the positions of strains PCB-180, SKT-109, and SSKT-41 are shown in red, while those of other DS-1-like G1P[8] strains are shown in blue. Bootstrap values of <75% are not shown. Scale bar: 0.05 substitutions per nucleotide.

The VP7 genes of strains PCB-180, SKT-109, and SSKT-41 exhibited the maximum nucleotide sequence identities (99.4–99.6%) with those of Japanese DS-1-like G1P[8] strains HC12016, NT004, and OH3506, and comparable identities (99.2%) with Thai human strain CU161-KK (G1P[8]) [24] and American human strain 2007719635 (G1P[8]). On phylogenetic analysis, strains PCB-180, SKT-109, and SSKT-41 were found to be closely related with strains HC12016, NT004, and OH3506 in a common branch with the above-mentioned G1P[8] strains, and Indonesian human strain YK-RVY36 (G1P[8]) and Paraguayan human strain 1701SR (G1P[9]) in G1 lineage-6 (Fig 3).

**Fig 4:**
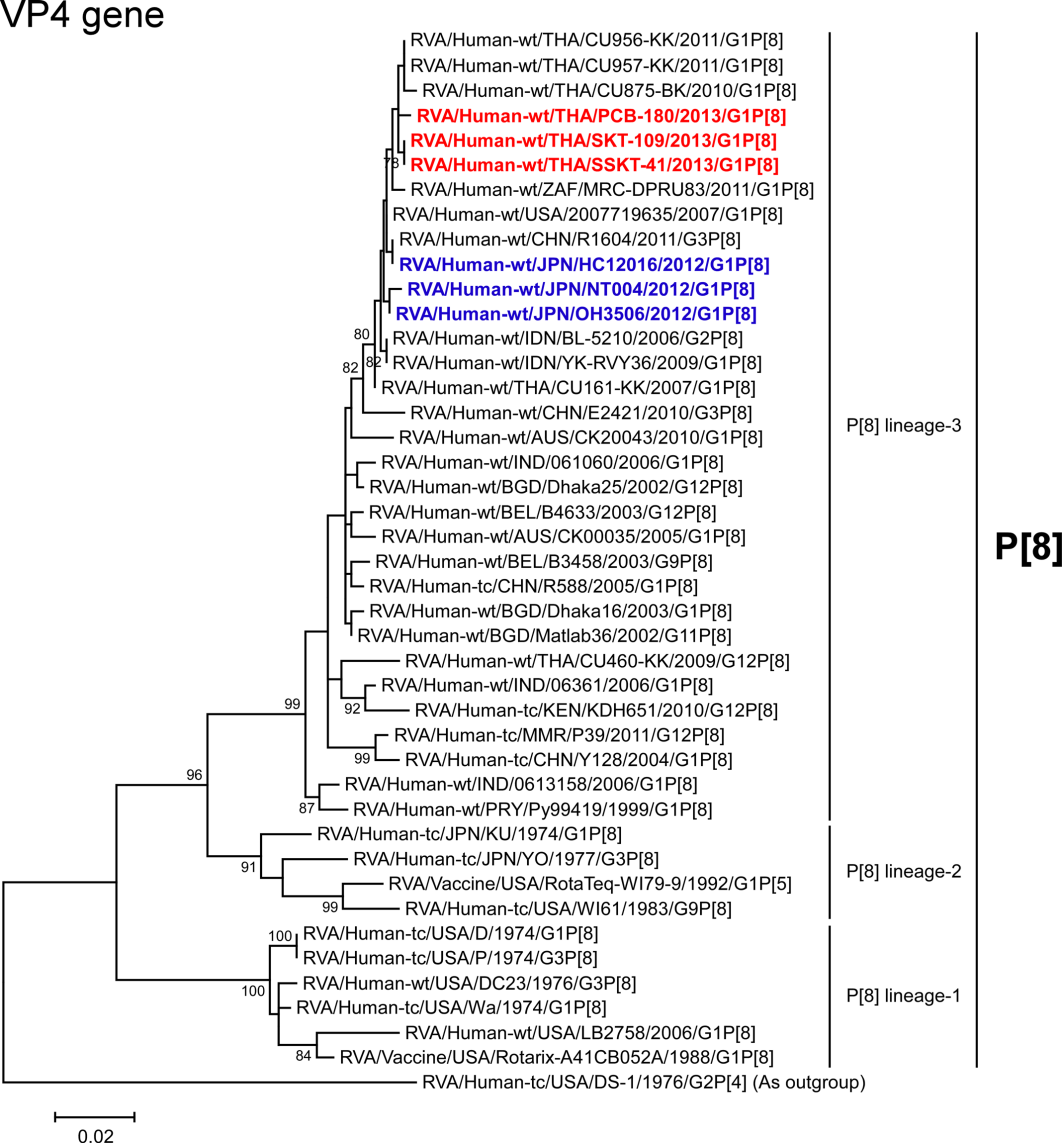
Phylogenetic tree constructed from the nucleotide sequences of the VP4 genes of strains PCB-180, SKT-109, and SSKT-41, and representative RVA strains. See legend of Fig 3. Scale bar: 0.02 substitutions per nucleotide.

The VP4 genes of strains PCB-180, SKT-109, and SSKT-41 showed the highest nucleotide sequence identities (99.5–99.6%) with the cognate genes of Thai human strain CU956-KK (G1P[8]) [25], Japanese DS-1-like G1P[8] strain OH3506, and American human strain 2007719635 (G1P[8]), and comparable identities (98.9–99.5%) with Thai human strains (CU957-KK (G1P[8]) and CU875-BK (G1P[8])) [25], Japanese DS-1-like G1P[8] strains (HC12016 and NT004), South African human strain MRC-DPRU83 (G1P[8]), and Chinese human strain R1604 (G3P[8]) [26]. On phylogenetic analysis, strains PCB-180, SKT-109, and SSKT-41 were shown to form a cluster near these strains in P[8] lineage-3 (Fig 4).

**Fig 5:**
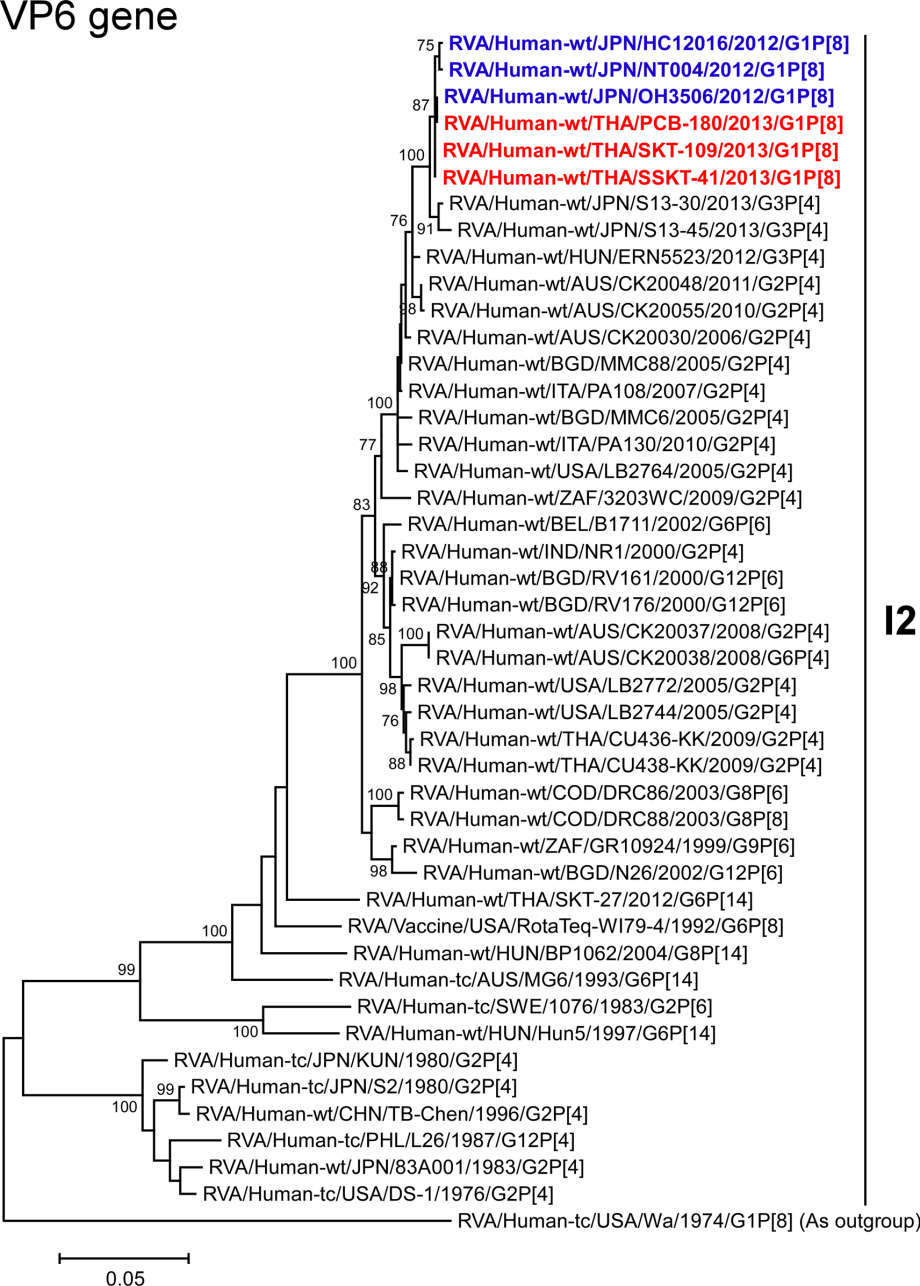
Phylogenetic tree constructed from the nucleotide sequences of the VP6 genes of strains PCB-180, SKT-109, and SSKT-41, and representative RVA strains. See legend of Fig 3. Scale bar: 0.05 substitutions per nucleotide.

The VP6 genes of strains PCB-180, SKT-109, and SSKT-41 exhibited the highest nucleotide sequence identities (99.7–100%) with the VP6 genes of Japanese DS-1-like G1P[8] strains HC12016, NT004, and OH3506, and somewhat lower identities (98.8–99.1%) with Japanese human strains (S13-30 (G3P[4]) and S13-45 (G3P[4])) [27], Australian human strains (CK20030 (G2P[4]) and CK20048 (G2P[4])), Italian human strains (PA108 (G2P[4]) and PA130 (G2P[4])) [28], Bangladeshi human strain MMC88 (G2P[4]) [29], and Hungarian human strain ERN5523 (G3P[4]) [30]. Phylogenetically, strains PCB-180, SKT-109, and SSKT-41 were closely related with strains HC12016, NT004, and OH3506 in a common branch with these strains (Fig 5).

**Fig 6:**
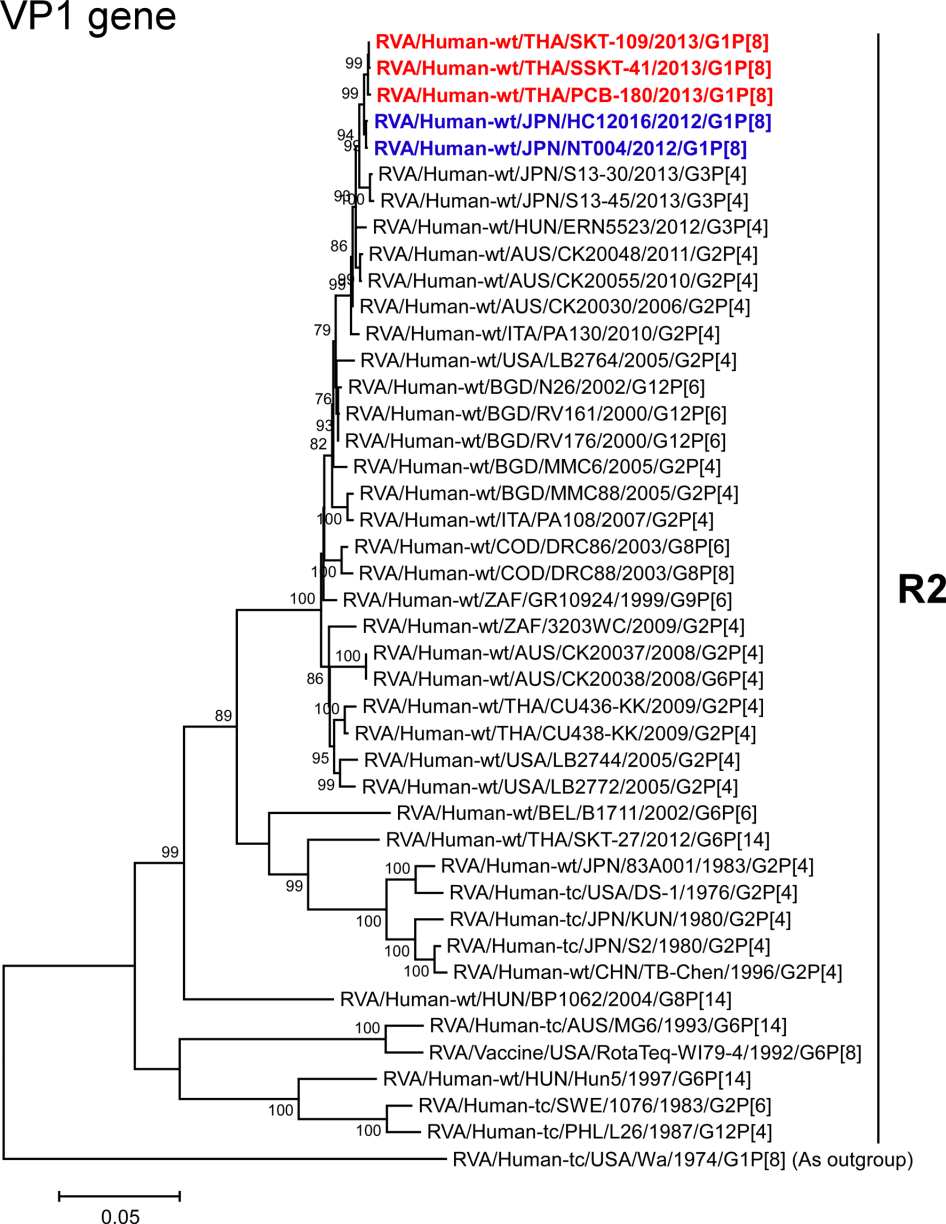
Phylogenetic tree constructed from the nucleotide sequences of the VP1 genes of strains PCB-180, SKT-109, and SSKT-41, and representative RVA strains. See legend of Fig 3. Scale bar: 0.05 substitutions per nucleotide.

**Fig 7:**
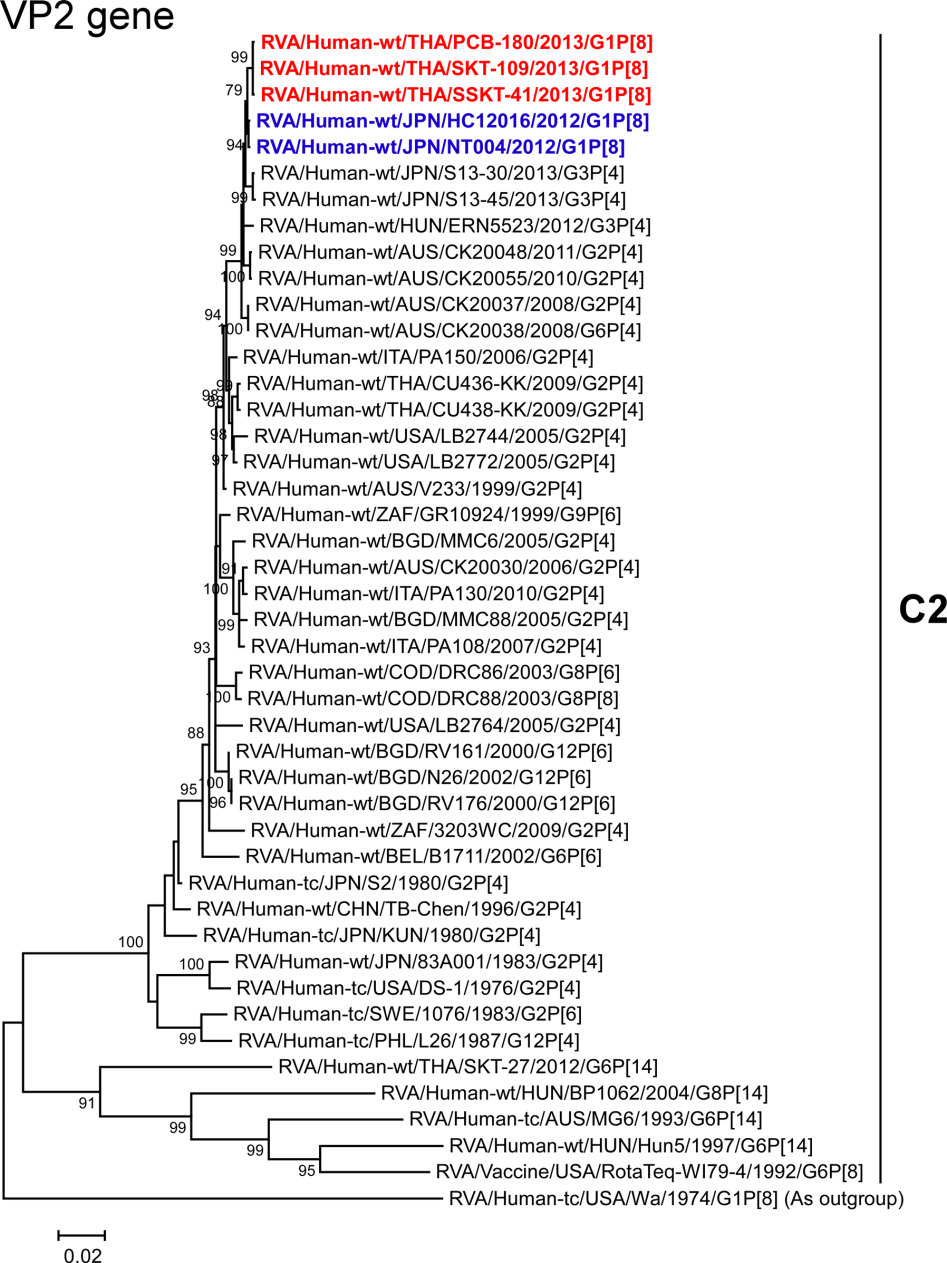
Phylogenetic tree constructed from the nucleotide sequences of the VP2 genes of strains PCB-180, SKT-109, and SSKT-41, and representative RVA strains. See legend of Fig 3. Scale bar: 0.02 substitutions per nucleotide.

**Fig 8:**
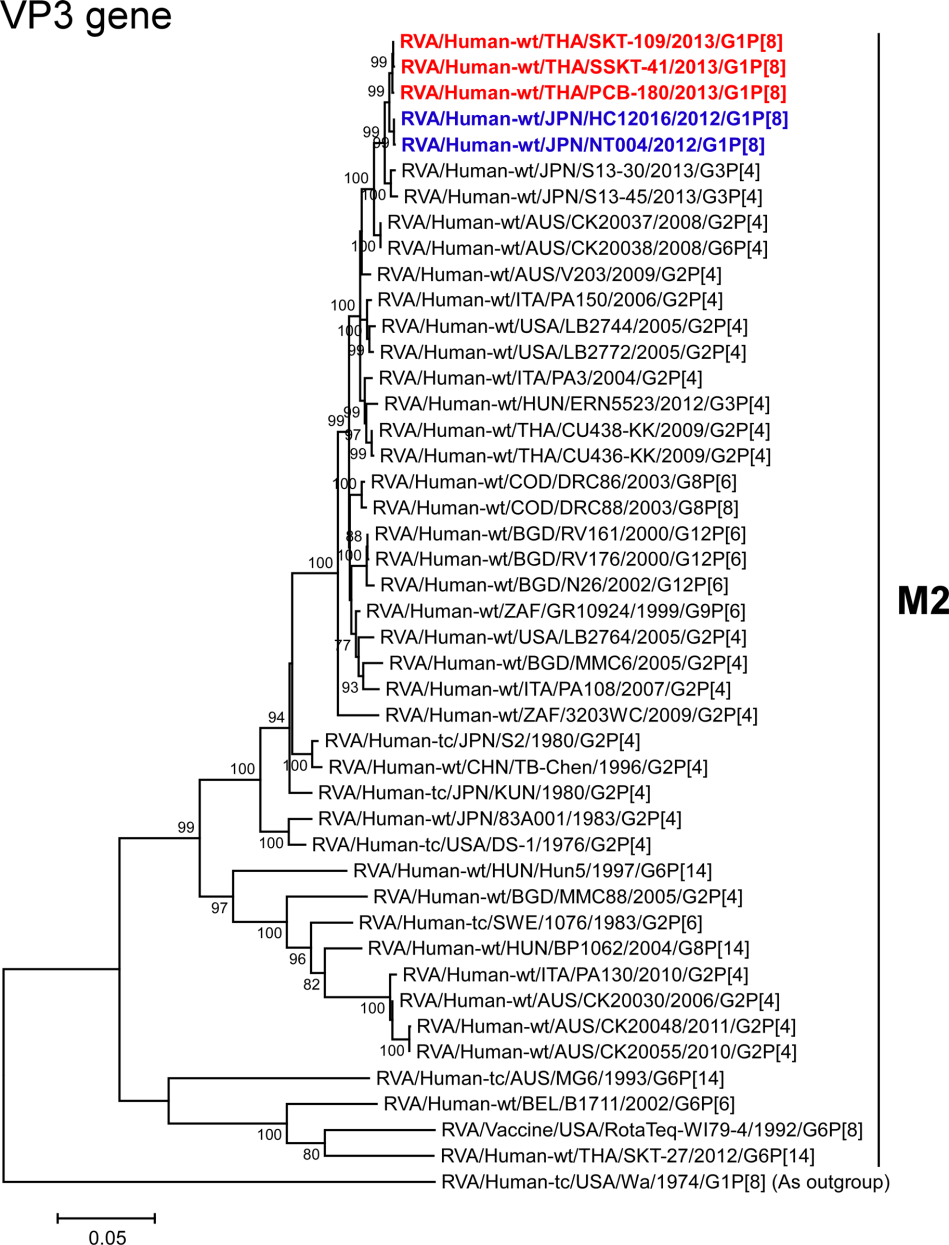
Phylogenetic tree constructed from the nucleotide sequences of the VP3 genes of strains PCB-180, SKT-109, and SSKT-41, and representative RVA strains. See legend of Fig 3. Scale bar: 0.05 substitutions per nucleotide.

**Fig 9:**
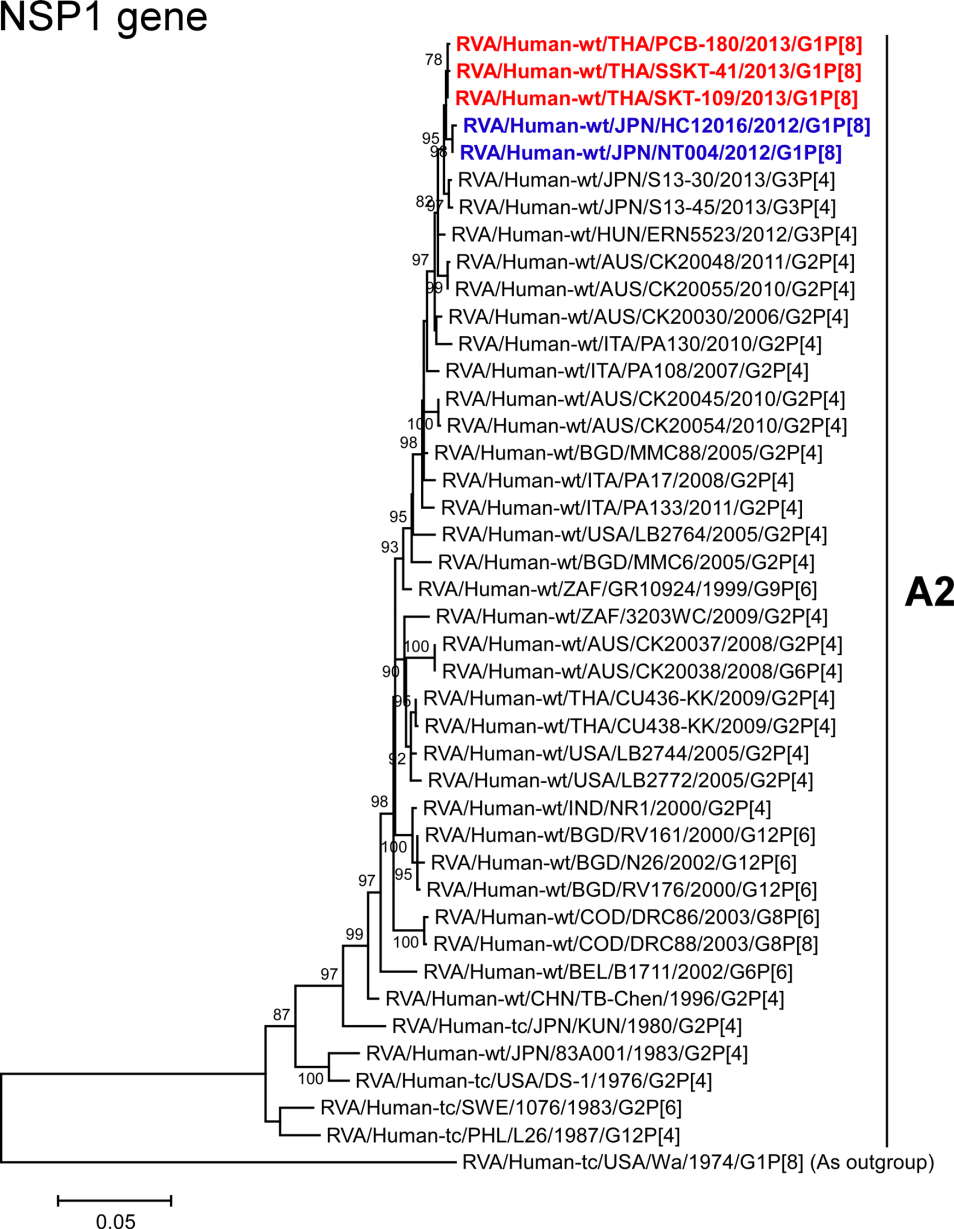
Phylogenetic tree constructed from the nucleotide sequences of the NSP1 genes of strains PCB-180, SKT-109, and SSKT-41, and representative RVA strains. See legend of Fig 3. Scale bar: 0.05 substitutions per nucleotide.

**Fig 10:**
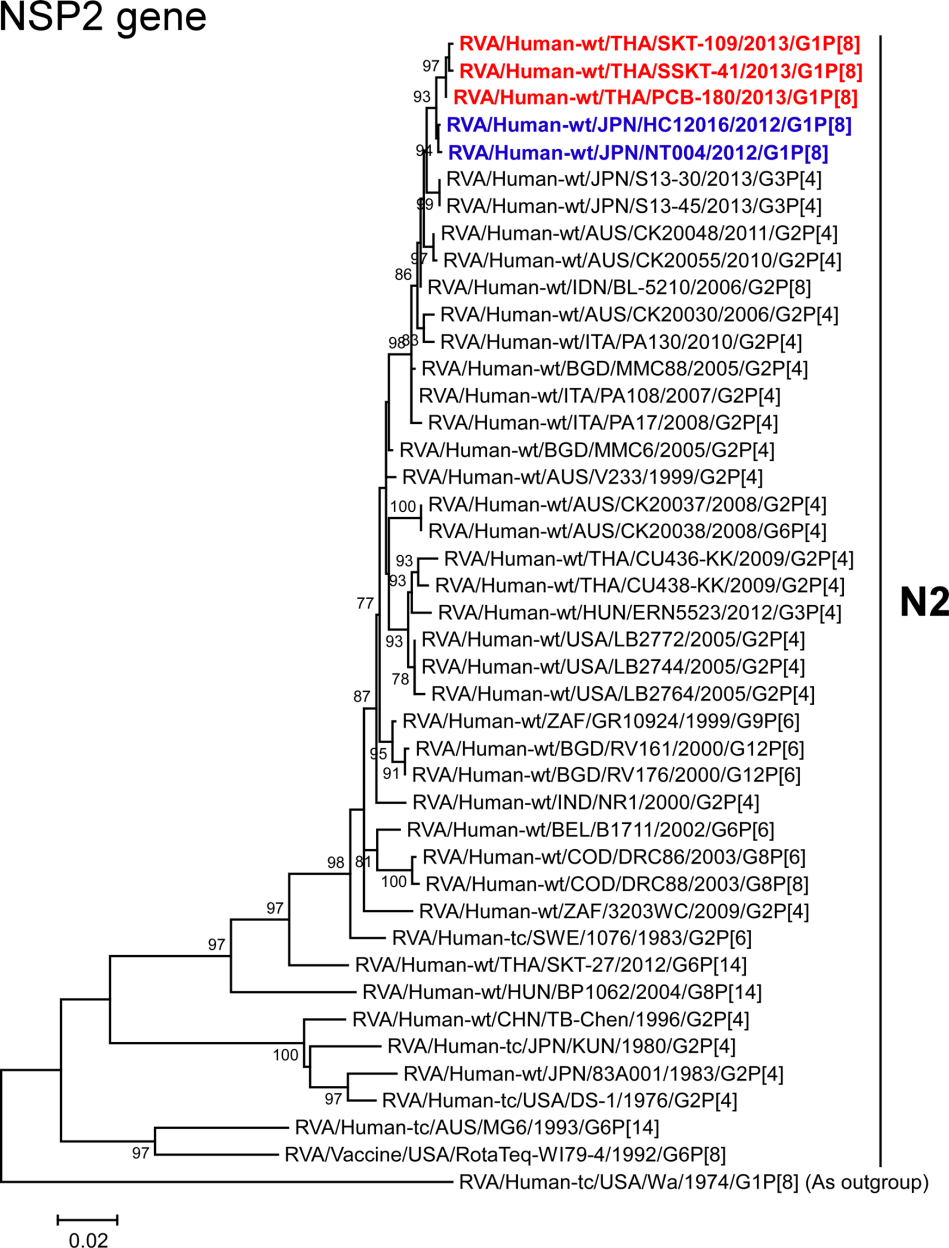
Phylogenetic tree constructed from the nucleotide sequences of the NSP2 genes of strains PCB-180, SKT-109, and SSKT-41, and representative RVA strains. See legend of Fig 3. Scale bar: 0.02 substitutions per nucleotide.

**Fig 11:**
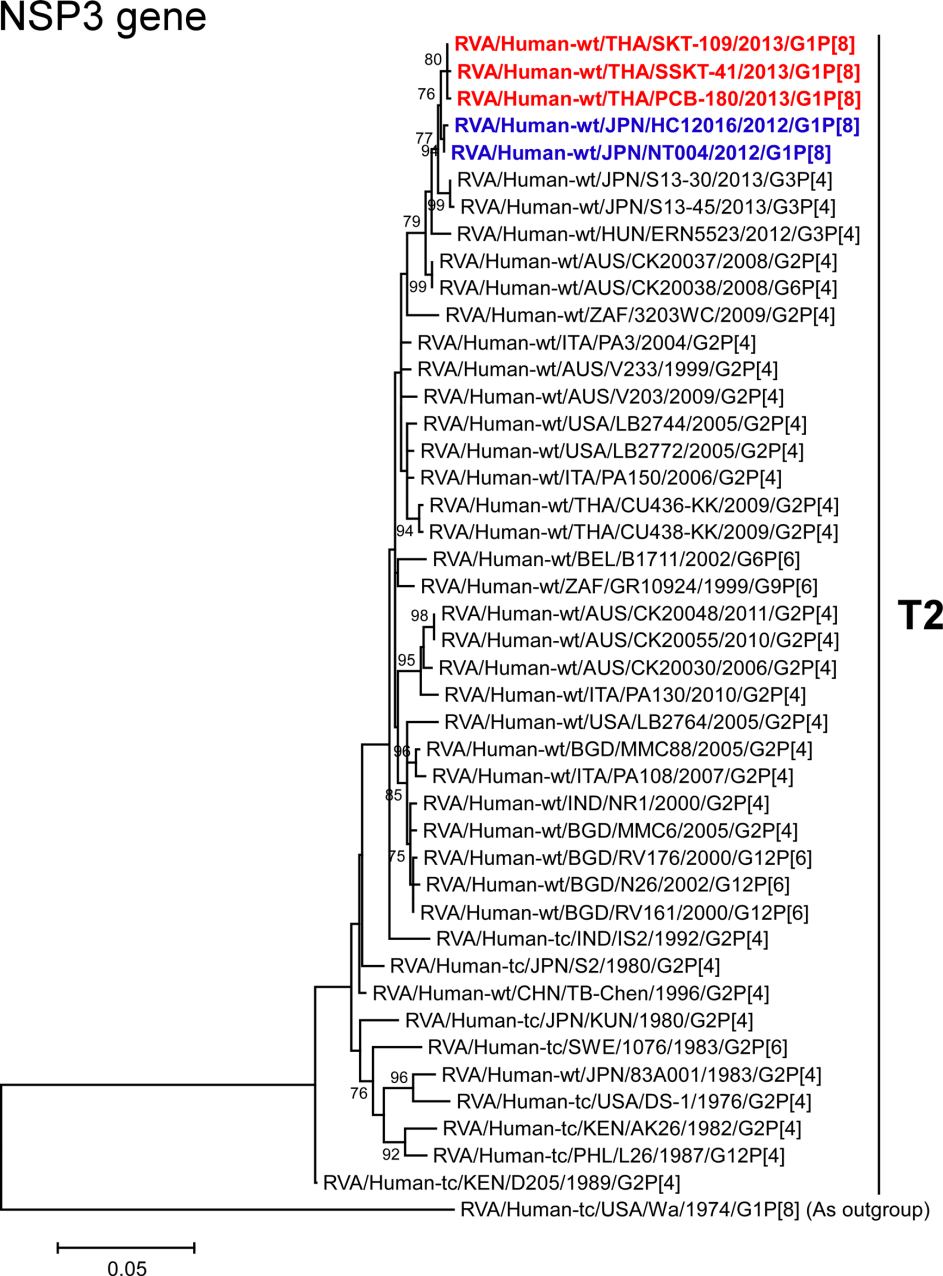
Phylogenetic tree constructed from the nucleotide sequences of the NSP3 genes of strains PCB-180, SKT-109, and SSKT-41, and representative RVA strains. See legend of Fig 3. Scale bar: 0.05 substitutions per nucleotide.

The VP1-3 and NSP1-3 genes of strains PCB-180, SKT-109, and SSKT-41 showed the maximum nucleotide sequence identities (99.6, 99.6–99.7, 99.5–99.6, 99.6–99.7, 99.4–99.6, and 99.4–99.7%, respectively) with those of Japanese DS-1-like G1P[8] strains HC12016 and NT004, and somewhat lower identities (98.9–99.1, 99.1–99.3, 98.5–99.0, 99.1–99.5, 98.7–99.2, and 98.8–99.1%, respectively) with several human strains from Australia, Hungary, Indonesia, Italy, and/or Japan. On phylogenetic analysis, strains PCB-180, SKT-109, and SSKT-41 were found to be closely related with strains HC12016 and NT004 in a common branch with these strains (Figs 6–11).

**Fig 12:**
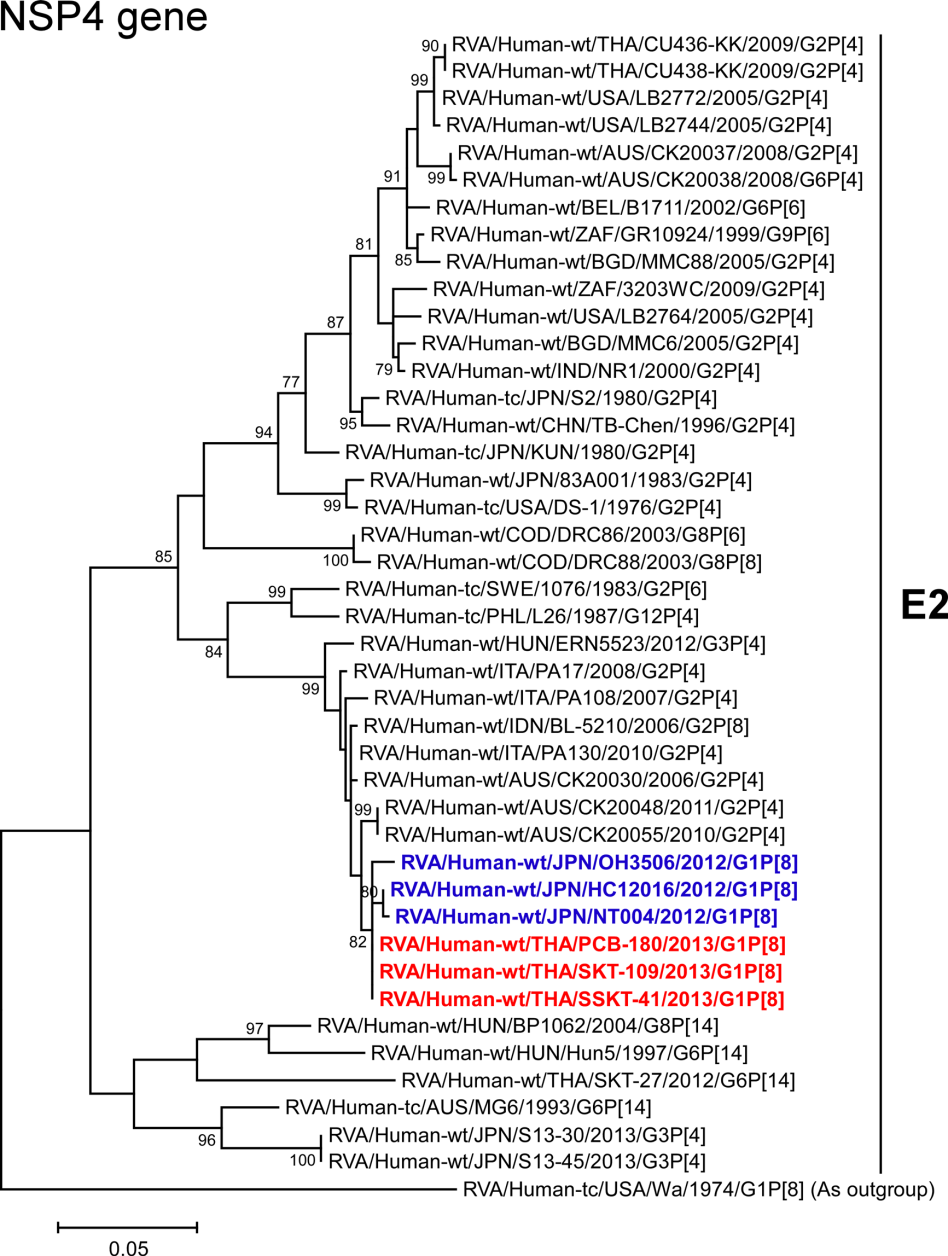
Phylogenetic tree constructed from the nucleotide sequences of the NSP4 genes of strains PCB-180, SKT-109, and SSKT-41, and representative RVA strains. See legend of Fig 3. Scale bar: 0.05 substitutions per nucleotide.

The NSP4 genes of strains PCB-180, SKT-109, and SSKT-41 showed the maximum nucleotide sequence identities (98.8–99.1%) with the cognate genes of Japanese DS-1-like G1P[8] strains HC12016, NT004, and OH3506, and comparable identities (98.4–98.6%) with Australian human strains (CK20030 (G2P[4]), CK20048 (G2P[4]), and CK20055 (G2P[4])), Indonesian human strain BL-5210 (G2P[4]), and Italian human strain PA130 (G2P[4]). On phylogenetic analysis, strains PCB-180, SKT-109, and SSKT-41 were closely related with strains HC12016, NT004, and OH3506 in a common branch with these strains (Fig 12).

**Fig 13:**
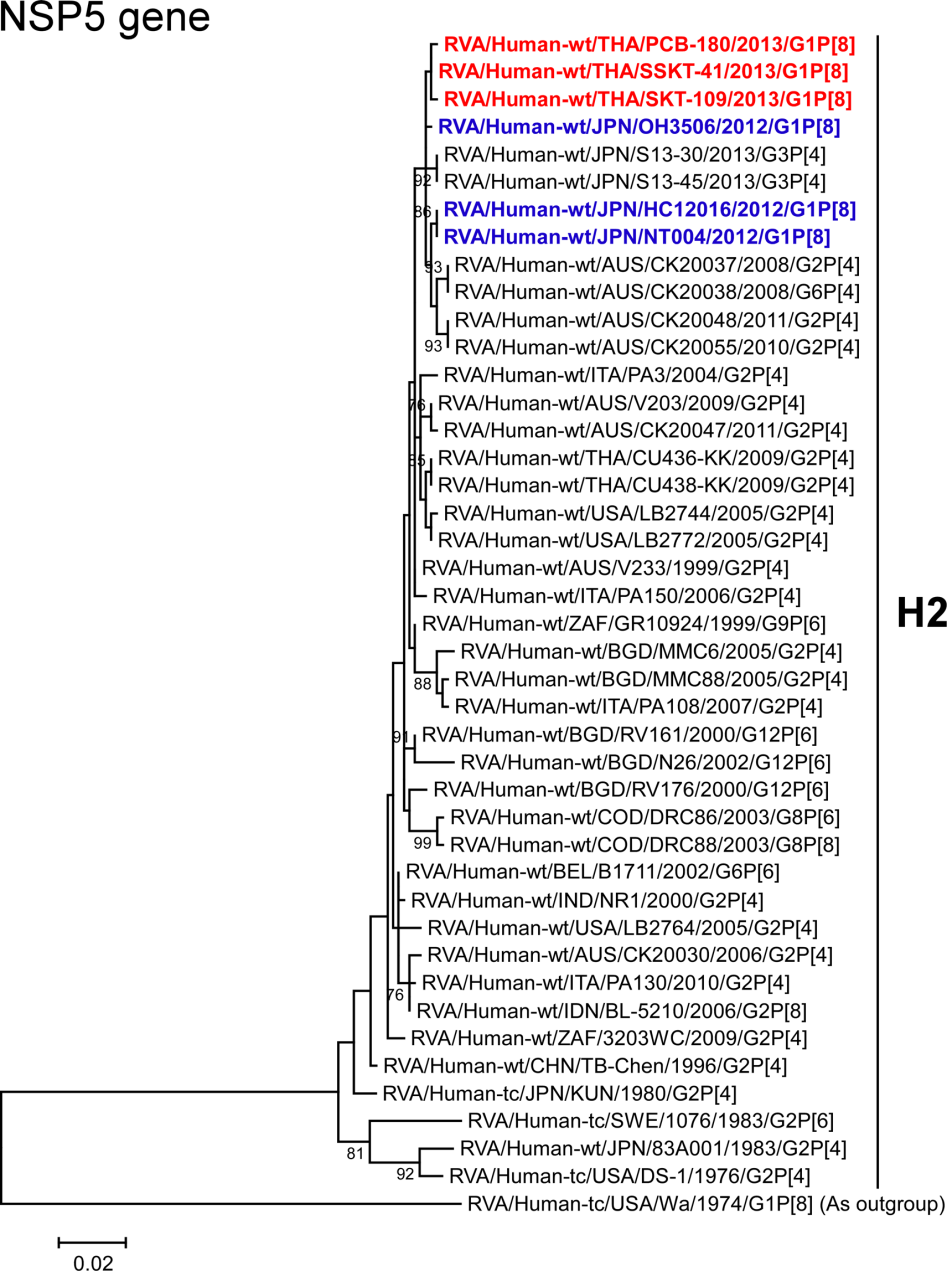
Phylogenetic tree constructed from the nucleotide sequences of the NSP5 genes of strains PCB-180, SKT-109, and SSKT-41, and representative RVA strains. See legend of Fig 3. Scale bar: 0.02 substitutions per nucleotide.

The NSP5 genes of strains PCB-180, SKT-109, and SSKT-41 exhibited the maximum nucleotide sequence identities (99.5–99.7%) with those of Japanese DS-1-like G1P[8] strains HC12016, NT004, and OH3506, and somewhat lower identities (98.2–99.2%) with Japanese human strains (S13-30 (G3P[4]) and S13-45 (G3P[4])) and Australian human strains CK20037 (G2P[4]), CK20038 (G6P[4]), CK20048 (G2P[4]), and CK20055 (G2P[4]). On phylogenetic analysis, strains PCB-180, SKT-109, and SSKT-41 were shown to be clustered near these strains (Fig 13).

In the present study, we analyzed the whole genomes of three DS-1-like G1P[8] strains that have emerged in Thailand (strains PCB-180, SKT-109, and SSKT-41) identified in stool specimens from hospitalized children with severe gastroenteritis during the RVA surveillance program, which involved a total of 687 RVA-positive stool samples. No difference in clinical presentation and severity was found among genotypes (Tacharoenmuang et al., in preparation). All the three Thai DS-1-like G1P[8] strains showed a unique genotype constellation including both genogroup 1 and 2 genes: G1-P[8]-I2-R2-C2-M2-A2-N2-T2-E2-H2. This unusual genotype constellation is shared with Japanese DS-1-like G1P[8] strains. Phylogenetic analysis revealed that the outer capsid genes (VP7 and VP4) of strains PCB-180, SKT-109, and SSKT-41 appeared to have originated from human Wa-like G1P[8] strains. On the other hand, the inner capsid and nonstructural genes (VP6, VP1-3, and NSP1-5) of these three strains were assumed to have originated from human DS-1-like strains. Therefore, strains PCB-180, SKT-109, and SSKT-41 were assumed to have been derived through reassortment event(s) between Wa-like G1P[8] and DS-1-like human rotaviruses. Furthermore, on phylogenetic analysis, strains PCB-180, SKT-109, and SSKT-41 were found to be very closely related to one another as to all the 11 genes, indicating the derivation of the three strains from a common origin. Moreover, all the 11 genes of these three strains were closely related with those of Japanese DS-1-like G1P[8] strains. Thus, DS-1-like G1P[8] strains that have emerged in Thailand and Japan appeared to have a common origin.

Notably, the VP4 genes of strains PCB-180, SKT-109, and SSKT-41 were closely related to those of Wa-like G1P[8] strains isolated in Thailand or the United States in 2007–2011. This could support the hypothesis that the reassortment event(s) between Wa-like G1P[8] and DS-1-like human strains occurred outside Japan, and the resultant DS-1-like G1P[8] strain was somehow introduced into Japan and then spread throughout the entire nation [10]. However, global rotavirus strain collection is required to determine the exact origin(s) of the DS-1-like G1P[8] strains. To our knowledge, this is the first description of full genome-based characterization of DS-1-like G1P[8] strains that have emerged in an area other than Japan. We need to continue surveying the emergence of such unusual RVA strains in the human population in order to determine the relationship with the introduction of rotavirus vaccines.

The emergence of DS-1-like G1P[8] strains in Thailand may imply the ongoing spread of these unusual RVA strains at least in Asia. Of note, DS-1-like G1P[8] strains have been successful in spreading and becoming predominant in broad locations across Japan [10, 12, 13]. Because it has not been examined as to whether or not the two available RVA vaccines (Rotarix (GlaxoSmithKline) and RotaTeq (Merck)) are still effective for prevention against unusual DS-1-like G1P[8] strains that share the G1P[8] genotype specificity with strains included in these vaccines, continuing RVA surveillance of DS-1-like G1P[8] strains is required. Although most studies on RVA genotype distributions have been focused on only G/P genes, PCR-based genotyping for non-G/P gene(s) or PAGE analysis should be performed to detect unusual RVA strains such as DS-1-like G1P[8] ones. Furthermore, whole genome-based analyses are essential to understand the evolutionary dynamics of emerging DS-1-like G1P[8] strains.

## Supporting Information

S1 Table Sequence data for the 11 gene segments of three Thai DS-1-like G1P[8] strains PCB-180, SKT-109, and SSKT-41. (DOCX)
