# Supplementary material for: Emergence and Characterization of Unusual DS-1-Like G1P[8] Rotavirus Strains in Children with Diarrhea in Thailand
Source: PLoS One. 2015 Nov 5;10(11):e0141739. doi: 10.1371/journal.pone.0141739 (PMC4634990; doi:10.1371/journal.pone.0141739)
Supplement: S1 Table — (DOCX) [file pone.0141739.s001.docx]

**S1 Table.** Sequence data for the 11 gene segments of three Thai DS-1-like G1P[8] strains PCB-180, SKT-109, and SSKT-41.

| Study strain | Total reads^a^ |  | Gene | | | | | | | | | | |
| --- | --- | --- | --- | --- | --- | --- | --- | --- | --- | --- | --- | --- | --- |
|  |  |  | VP7 | VP4 | VP6 | VP1 | VP2 | VP3 | NSP1 | NSP2 | NSP3 | NSP4 | NSP5 |
| RVA/Human-wt/THA/PCB-180/2013/G1P[8] | 1,402,222 | Nucleotide (bp) | 1050 | 2350 | 1340 | 3290 | 2673 | 2575 | 1560 | 1047 | 1063 | 744 | 794 |
|  |  | Deduced amino acids (aa) | 326 | 775 | 397 | 1088 | 879 | 835 | 486 | 317 | 310 | 175 | 200 |
|  |  | Reads mapped to gene segment | 82,513 | 124,596 | 31,604 | 199,888 | 165,793 | 134,715 | 49,730 | 67,180 | 60,894 | 15,492 | 6,636 |
|  |  | Maximum depth of reads | 16,200 | 9,893 | 7,529 | 13,662 | 9,418 | 7,169 | 10,286 | 10,567 | 7,506 | 6,438 | 1,817 |
| RVA/Human-wt/THA/SKT-109/2013/G1P[8] | 849,338 | Nucleotide (bp) | 1062 | 2359 | 1356 | 3298 | 2684 | 2591 | 1566 | 1049 | 1067 | 746 | 807 |
|  |  | Deduced amino acids (aa) | 326 | 775 | 397 | 1088 | 879 | 835 | 486 | 317 | 310 | 175 | 200 |
|  |  | Reads mapped to gene segment | 57,628 | 116,271 | 67,637 | 157,824 | 120,373 | 102,289 | 44,345 | 64,580 | 51,902 | 27,401 | 24,572 |
|  |  | Maximum depth of reads | 9,260 | 9,630 | 7,183 | 8,084 | 5,674 | 5,575 | 6,237 | 12,559 | 6,107 | 5,839 | 6,183 |
| RVA/Human-wt/THA/SSKT-41/2013/G1P[8] | 1,468,588 | Nucleotide (bp) | 1062 | 2359 | 1356 | 3302 | 2684 | 2591 | 1566 | 1049 | 1066 | 745 | 813 |
|  |  | Deduced amino acids (aa) | 326 | 775 | 397 | 1088 | 879 | 835 | 486 | 317 | 310 | 175 | 200 |
|  |  | Reads mapped to gene segment | 40,999 | 55,325 | 17,761 | 117,117 | 105,880 | 81,361 | 36,517 | 62,342 | 44,691 | 13,330 | 9,247 |
|  |  | Maximum depth of reads | 5,369 | 4,337 | 6,447 | 5,530 | 8,064 | 4,107 | 6,355 | 9,903 | 4,963 | 5,619 | 2,848 |

^a^Sequence reads remaining after adapter trimming.
